# Pathogenesis-based preexposure prophylaxis associated with a low risk of SARS-CoV-2 infection in healthcare workers at a designated COVID-19 hospital: a pilot study

**DOI:** 10.1186/s12879-021-06241-1

**Published:** 2021-06-07

**Authors:** Michael V. Dubina, Veronika V. Gomonova, Anastasia E. Taraskina, Natalia V. Vasilyeva, Sergey A. Sayganov

**Affiliations:** 1 State Research Institute of Highly Pure Biopreparations FMBA Russia, 7 Pudozhskaya str, St. Petersburg, 197110 Russia; 2 Russian Academy of Sciences, 14 Leninskiy pr, 119991 Moscow, Russia; 3 North-Western State Medical University named after I.I. Mechnikov of Ministry of Health of Russian Federation, 41 Kirochnaya str, 191015 St. Petersburg, Russia

**Keywords:** COVID-19, Coronavirus disease, SARS-CoV-2, Preexposure prophylaxis, PrEP

## Abstract

**Background:**

At present, no agents are known to be effective at preventing COVID-19. Based on current knowledge of the pathogenesis of this disease, we suggest that SARS-CoV-2 infection might be attenuated by directly maintaining innate pulmonary redox, metabolic and dilation functions using well-tolerated medications that are known to serve these functions, specifically, a low-dose aerosolized combination of glutathione, inosine and potassium.

**Methods:**

From June 1 to July 10, 2020, we conducted a pilot, prospective, open-label, single-arm, single-center study to evaluate the safety and efficacy of preexposure prophylaxis (PrEP) with aerosolized combination medication (ACM) on the incidence of SARS-CoV-2 positivity in 99 healthcare workers (HCWs) at a hospital designated for treating COVID-19 patients. We compared SARS-CoV-2 positivity in ACM users to retrospective data collected from 268 untreated HCWs at the same hospital. Eligible participants received an aerosolized combination of 21.3 mg/ml glutathione and 8.7 mg/ml inosine in 107 mM potassium solution for 14 days. The main outcome was the frequency of laboratory-confirmed SARS-CoV-2 cases, defined as individuals with positive genetic or immunological tests within 28 days of the study period.

**Results:**

SARS-CoV-2 was detected in 2 ACM users (2, 95% CI: 0.3 to 7.1%), which was significantly less than the incidence in nonusers, at 24 (9, 95% CI: 5.8 to 13.0%; *P* = 0.02). During the PrEP period, solicited adverse events occurred in five participants; all were mild and transient reactions.

**Conclusions:**

Our findings might be used either to prevent SARS-CoV-2 infection or to support ongoing and new research into more effective treatments for COVID-19.

**Trial registration:**

ISRCTN, ISRCTN34160010. Registered 14 September 2020 - Retrospectively registered.

**Supplementary Information:**

The online version contains supplementary material available at 10.1186/s12879-021-06241-1.

## Background

COVID-19, a novel infection caused by severe acute respiratory syndrome coronavirus 2 (SARS-CoV-2), was first detected in humans in December 2019 [1]. As of May 2, 2021, 150 million confirmed cases and 3 million deaths have been reported worldwide [2]. The clinical hallmarks of the disease vary from asymptomatic infection to pneumonia featuring ground-glass opacities that might progress to life-threatening complications such as acute respiratory distress syndrome (ARDS) and multisystem organ failure [3]. Older patients and those with preexisting respiratory, cardiovascular and metabolic conditions appear to be at the greatest risk for severe complications and death [4]. Healthcare workers (HCWs) are at particularly high risk of acquiring SARS-CoV-2 due to repeated exposure to infected patients [5, 6]. A notable genomic feature of SARS-CoV-2 is its long spike (S) glycoprotein, which shows approximately 80% sequence identity with the S proteins of the viruses SARS-CoV and MERS [7]. Based on the similarities and past studies of SARS-CoVs, the pathogenesis of COVID-19 likely includes phases of SARS-CoV-2 infection that correspond to viral entry, replication in the upper airway and migration down the respiratory tract, with a robust immune response that triggers hypoxia and progression to ARDS [8]. In the absence of a proven effective therapy, off-label or compassionate-use compounds have been used to treat COVID-19 with the assumption that airway epithelium damage, a diverse immune response and inflammation predominate in SARS-CoV-2 pathogenicity [9]**.** Although there are currently almost three hundred COVID-19 candidate vaccines in development [10], to date, no agents are known to be effective in preventing SARS-CoV-2 infection [11].

Angiotensin-converting enzyme 2 (ACE2) has been identified to act as a receptor and an entry point for SARS-CoV-2, similar to what has been previously identified for SARS-CoVs [12]. However, structural, biochemical and modeling data reveal that SARS-CoV-2 might recognize ACE2 with a binding affinity that is an order of magnitude greater than that of SARS-CoV [13, 14]**.** Although generally low ACE2 expression is found in airway cells [15], ACE2 is markedly expressed in nasal and alveolar epithelial cells and is present in arterial smooth muscle cells and endothelial cells [16, 17]. ACE2 represses the production of angiotensin II (Ang II) by angiotensin-converting enzyme [18]**.** Previous studies have reported considerably reduced ACE2 expression in the lungs of wild-type mice infected with SARS-CoV, suggesting that ACE2 might have a role in SARS-CoV-mediated severe acute lung pathologies [19]. When SARS-CoVs interact with ACE2 to gain entry into cells, downregulation of ACE2 – either directly due to viral binding or indirectly due to cell lysis – decreases Ang II inhibition [20]. Therefore, SARS-CoV-2 can reduce ACE2 activity and receptor consumption, further exacerbating an ACE2/Ang II regulatory imbalance [21] and resulting in pulmonary vasoconstriction [22] and impaired metabolic homeostasis in smooth muscle cells (SMCs) [23] of distal bronchiolar airways.

Taken together, the key role of ACE2 in SARS-CoV-2 infection, the strong binding affinity of SARS-CoV-2 to the ACE2 receptor, and the documented severity of COVID-19 in subjects with respiratory, cardiovascular and metabolic conditions led us to hypothesize that pulmonary vasoconstriction and impaired airway SMC metabolic homeostasis resulting from robust SARS-CoV-2 virus-induced ACE2/Ang II regulatory imbalance might prevail over the diverse immune response at the initial phase of COVID-19 pathogenesis. Although pulmonary vasoconstriction is a physiological mechanism by which the pulmonary arteries maintain blood oxygenation during alveolar hypoxia [24], Ang II can induce robust pulmonary vasoconstriction via angiotensin receptor 1 (AT1R) [25]. Ang II can also cause internalization and degradation of membrane Ca^2+^-activated K^+^ channels (BKCa), providing an additional mechanism contributing to vasoconstriction [26]. Moreover, hypoxia, per se*,* might act either directly on peripheral pulmonary arterial SMCs to inhibit voltage-sensitive K^+^ channels (Kv) and induce membrane depolarization and contraction [27] or indirectly to stimulate vasoconstrictor release and/or inhibit vasodilator release [28]. Genome-wide transcriptome analyses in vivo after Ang II infusion have revealed upregulation of genes involved in metabolism and ion transport pathways, whereas genes that are protective against oxidative stress, including glutathione synthetase and mitochondrial superoxide dismutase 2, were downregulated [29]. Overall, disruption of reduction-oxidation (redox) signaling and excessive generation of reactive oxygen species by the injured pulmonary endothelium/epithelium under pathological conditions lead to increased endothelial permeability and enhanced expression of proinflammatory cytokines and adhesion molecules, amplifying tissue damage and pulmonary edema [30]. Therefore, we hypothesized that SARS-CoV-2 infectivity and pathogenicity and the resulting pulmonary abnormalities induced by an ACE2/Ang II regulatory imbalance might be attenuated by directly maintaining the innate redox, dilation and metabolic functions of the lung using appropriate and well-tolerated medications.

Safe medications that have been previously reported to maintain ventilation-perfusion functions of the lung are currently available. A prime example is glutathione (γ-l-glutamyl-l-cysteinyl-glycine, GSH), a major nonprotein thiol [31], the levels of which decrease in alveolar epithelial cells upon exposure to toxins or respiratory viruses [32]; this decrease is associated with increased superoxide production and proinflammatory cytokine release [33]. It has been reported that GSH can be delivered via aerosol to directly augment the GSH level in the epithelial lining fluid of the lower respiratory tract in vivo [34] and to improve clinical outcome in patients with cystic fibrosis [35]. GSH treatment of isolated bronchi in vitro resulted in decreased SMC contraction and bronchodilation [36], increasing membrane hyperpolarization via potassium (K+) channels on airway SMCs [37]. In the human lung, Kv and BKCa channels located in the apical membrane contribute to a high K+ content in the adult airway surface liquid [38], and extracellular K+ is involved in matching tissue blood flow as a mediator of functional vasodilation [39]. Moreover, vasodilators that act through receptors coupled to guanine nucleotide-binding protein (G-protein) activate K+ channels through the cAMP signaling cascade, which includes adenosine [40]. Hydrolytic deamination of adenosine generates inosine, which increases in the extracellular space under metabolically stressful conditions and has been shown to have powerful immunomodulatory and cytoprotective activities by binding to G-protein-coupled A2A adenosine receptors [41]. Furthermore, inosine might serve as an alternative substrate for ATP generation during hypoxia [42] and protects the bronchial and alveolar epithelium from the potentially deleterious consequences of neutrophil accumulation [43]. Inosine might also exert antiviral effects through incorporation into double-stranded viral RNA and potentiation of immune system sensing [44]. It is important to note that inosine-based compounds are among the drugs that are being repurposed for the management of COVID-19 [45]; in addition, the efficacy of high-dose glutathione therapy in relieving the dyspnea associated with COVID-19 pneumonia has recently been reported [46].

Based on the high risk of infection in this population, the rationale presented above and the absence of effective agents to prevent or treat COVID-19, we aimed to evaluate whether preexposure prophylaxis (PrEP) using a low-dose aerosolized combination of glutathione, inosine and potassium might prevent SARS-CoV-2 infection in healthy adults who are at high risk for exposure to the virus.

## Methods

### Design

A pilot, prospective, longitudinal cohort, open-label study with retrospective control was designed and conducted from June 1, 2020, to July 10, 2020. The study was initiated and conducted at North-Western State Medical University named after I.I. Mechnikov (NWSMU), a large healthcare center with 100 clinics and 4500 employees. The study population was defined as healthy individuals who deliver care and services to COVID-19 patients (healthcare workers, HCWs), either directly as physicians or nurses or indirectly as assistants, technicians, or other support staff, while using mandatory personal protective equipment in accordance with national guidelines. Healthy volunteers were recruited from among HCWs at a 264-bed hospital in NWSMU that was governmentally designated to treat COVID-19 patients as of May 5, 2020. Full details of the study design and conduct are provided in a protocol (Additional file 1).

### Participants

**Fig. 1:**
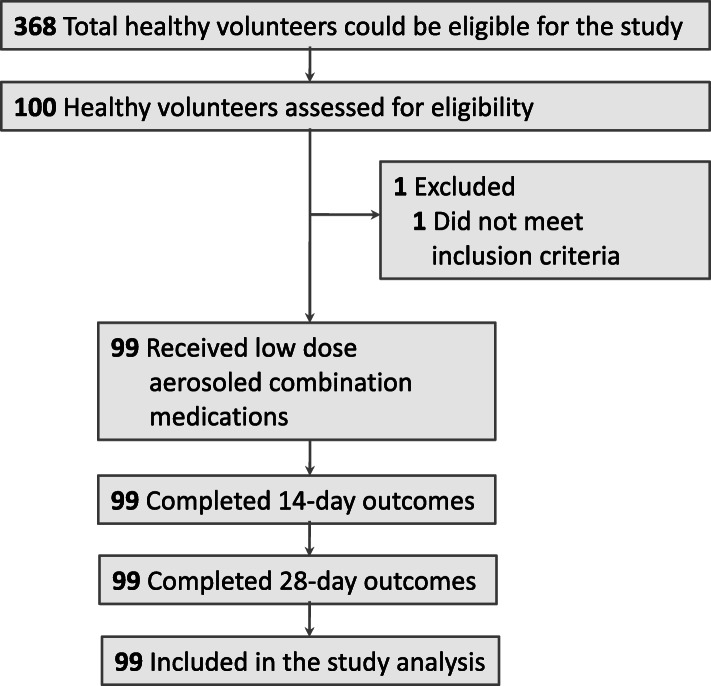
Flow diagram showing the progression of healthy subjects throughout the study

Given the rapid emergence and evolving situation regarding the COVID-19 pandemic, limited data were available at the time of study design on which to base sample size estimates. Based on preliminary observational trials in high-risk individuals routinely exposed to COVID-19-positive contacts, the frequency of confirmed SARS-CoV-2 infection is not less than 11% [5, 6]. We expected a significant decrease to 3% or less of SARS-CoV-2-positive cases during the study period, with sample size of 96 or more healthy volunteers in the PrEP treatment group based on an alpha error rate of 0.05 and a beta error rate of 0.85. In this scenario, the number needed to treat (NNT) is 13. In other words, we would have to treat 13 healthy people for one individual to have been saved from SARS-CoV-2 infection. We used a voluntary response sampling method to recruit participants for this prospective cohort longitudinal study; recruitment was performed by members of the study team at the hospital. From the full staff of 410 HCWs at the hospital, 100 HCWs were assigned to the study based on their negative genetic and immunological results at the end of May. All participants completed a pre-enrollment evaluation, which included data from genetic and serological tests for SARS-CoV-2 performed prior to enrollment. One of the participants tested positive for SARS-CoV-2 based on the PCR of his swab sample, which was taken before the start of the study. This participant was excluded from the study in accordance with the exclusion criteria in the protocol. From June 1 to June 12, 2020, 99 RNA- and antibody-negative healthy volunteers were assigned to this study (Fig. 1). Data on treatment adherence and adverse events were collected on day 7 and day 14; follow-up information was solicited through day 28 in accordance with the protocol. The NWSMU collected the data and monitored the program.

### Intervention

We used two solutions to prepare a low-dose aerosolized combination of three medications; the two solutions were 3% inosine-glutamyl-cysteinyl-glycine disodium (i.e., inosine-glutathione; Molixan, PharmaVAM; St. Petersburg, Russia) – a patented metabolic agent that is approved in Russia for parenteral use for combination treatment of viral hepatitis (grls.rosminzdrav.ru, N001355/02), and 4% potassium chloride (Solopharm, St. Petersburg, Russia). The medication for each 5-min inhalation session was prepared ex tempore by the participants themselves by mixing solutions of 1.0 ml inosine-glutathione and 0.25 ml potassium chloride to achieve final concentrations of 21.3 mg/ml glutathione, 8.7 mg/ml inosine and 107 mM potassium (pH 5.5). The combined medication was self-administered as an aerosol using a personal handheld nebulizer (Nebzmart MBPN002; MicroBase Technology, Taiwan) driven by compressed air at 0.25 ml/min. Eligible participants received the study treatment for 14 days; they were instructed to perform four inhalation sessions per day, with 4 h in between sessions.

### SARS-CoV-2 testing

Nasopharyngeal swabs were collected for genetic detection of SARS-CoV-2 by real-time polymerase chain reaction (PCR), and venous blood draws were performed for immunological assessments. All samples were deidentified, transported to the NWSMU diagnostic laboratory within 3 h, and stored at + 4 °C until analysis. Total RNA was extracted from the swabs using an RNA-Express Kit (Lytech, Moscow, Russia). SARS-CoV-2 was tested by PCR using two target genes, namely, the RNA-dependent RNA polymerase (RdRp) gene of the open reading frame 1ab (ORF1ab) sequence and the envelope (E) gene, and the POLIVIR SARS-CoV-2 Assay (Lytech, Moscow, Russia) with the CFX-96 Touch system (Bio-Rad, Hercules, USA). A result was considered positive if the cycle threshold was below 35 for both target genes. Serum antibodies were assayed using two diagnostic kits approved in Russia for qualitative detection of immunoglobulins against SARS-CoV-2 in human plasma, per the manufacturers’ instructions: SARS-CoV-2-IgМ-IFA BEST (Vector-Best, Novosibirsk, Russia) and SARS-CoV-2–IgG (Lytech, Moscow, Russia). Antibodies were measured as the optical density at 450 nm using a HumaReader HS microtiter plate reader (HUMAN, Wiesbaden, Germany).

### Outcome

The primary outcome was new-onset SARS-CoV-2 infection, as detected by genetic or immunological tests over the study period. All HCWs in the hospital were routinely tested for SARS-CoV-2 once per week in accordance with the national guidelines and recommendations: a nasopharyngeal swab was collected and analyzed with PCR for SARS-CoV-2. HCWs with positive SARS-CoV-2 PCR results remained on sick leave until a negative follow-up PCR was obtained. They were allowed to return to work when the symptoms had resolved, and they had a negative follow-up PCR. Additionally, all HCWs in the hospital underwent qualitative serological testing for IgM and IgG against SARS-CoV-2 at the end of May to exclude anyone with an asymptomatic infection before the start of the study on June 1, 2020, and at the beginning of July 2020 to detect individuals who became infected during the study. SARS-CoV-2 positivity was defined as the presence of RNA, IgM or/and IgG. We used all available data and did not separate RNA, IgM and IgG positivity to confirm the total SARS-CoV-2 incidence in HWCs at high risk of infection. A comparison group of HCWs from the hospital was analyzed retrospectively; these HCWs were not included in the study group, were RNA- and antibody-negative at the end of May 2020 and worked until the final date of the study. The outcome data in the comparison group were obtained anonymously from routine COVID-19 surveillance reports at the hospital.

### Statistical analysis

The chi-square test was used to compare the incidence of SARS-CoV-2 positivity between the study group and the comparison group; a *p*-value less than 0.05 was considered statistically significant. Because the analysis did not include a provision for correcting for multiple comparisons in tests for association between baseline variables and outcomes, the results are reported as point estimates and 95% confidence intervals. The analysis was conducted using SAS software version 9.4 M6 University Edition (2018).

### Trial registration

ISRCTN, ISRCTN34160010. Registered 14 September 2020 - Retrospectively registered, http://www.isrctn.com/ISRCTN34160010.

## Results

During the study period (June 1, 2020, to July 10, 2020), the cumulative number of patients with COVID-19 in the hospital increased from 447 to 884 (97.8%). Among the 410 HCWs who worked at the hospital, 43 (10.5%) were not included in the study: 29 (7.1%) were SARS-CoV-2 positive before the beginning of the study, and 14 (3.4%) did not work until the final date of the study. In total, 99 HCWs were enrolled as participants in the study, comprising the ACM group (24.1%); the remaining 268 HCWs were designated as the comparison group (65.4%).

The mean age of the participants was 27.0 years (95% CI: 25.3 to 28.7), 69% were female, and 52% were nurses (51/99). The demographic characteristics of the participants and the comparison group did not differ significantly (Table 1).

**Table 1:** Baseline characteristics of HCWs in a COVID-19 hospital

Characteristic	ACM users (***n*** = 99)	Nonusers (***n*** = 268)
Demographic		
Median age – yrs. (IQR)	25 (5)	25 (11)
Age category – No. (%)		
< 40	88 (89)	228 (85)
40 to 65 yrs	11 (11)	40 (15)
Male–No. (%)	31 (31)	106 (40)
Profession		
Physicians – No. (%)	38 (38)	99 (37)
Nurses – No. (%)	51 (52)	140 (52)
Others – No. (%)	10 (10)	29 (11)

*HCWs* Healthcare workers, *ACM* Aerosolized combination medication, *IQR* Interquartile range

**Fig. 2:**
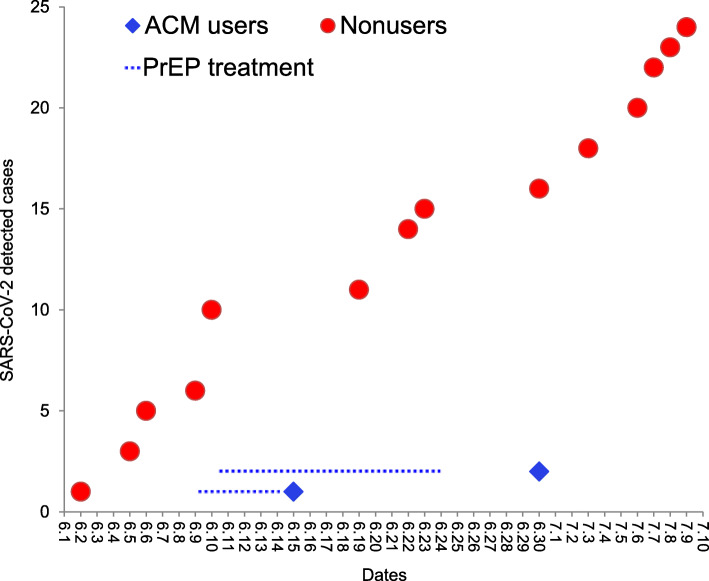
Cumulative SARS-CoV-2 incidence in HCWs. HCWs – healthcare workers. ACM – aerosolized combination medication. PrEP – preexposure prophylaxis with ACM

The total incidence of positivity for SARS-CoV-2 detected in the ACM group was 2% (2/99; 95% CI: 0.3 to 7.1%), which was significantly less than the RNA- or antibody-positivity rate in the comparison group, which was 9% (24/268; 95% CI: 5.8 to 13.0%; *P* = 0.02) (Fig. 2). The NNT for the PrEP treatment in our study was 14.

During the study period, no serious adverse events were observed in the ACM group, and none of the stopping rules prespecified in the study protocol were met. During the treatment period, solicited systemic and common adverse events, which occurred in five participants (5%), included headache in two (2%), itchy throat in two (2%) and dry cough in one (1%). All adverse events were mild and transient reactions lasting no longer than 30 min during or after the inhalation session. Indeed, no patterns of concern were observed for the adverse events, and neither treatment interruption nor modification was indicated.

## Discussion

Here, we report that pathogenesis-based prophylaxis was associated with a significant reduction in SARS-CoV-2 positivity incidence, to 2%, in HCWs who delivered care and services to patients with COVID-19 compared with a 9% incidence in the remaining HCWs who did not use the treatment at the same hospital during the entire study period. Effective PrEP for COVID-19 would not only safely prevent high-risk individuals from acquiring SARS-CoV-2 infection from close contacts infected with the virus but may also attenuate the infection if it does occur. At present, most clinical trials of pharmacologic interventions to prevent SARS-CoV-2 infection have been conducted with medications previously used for the treatment of COVID-19 (e.g., hydroxychloroquine) and have focused on evaluating the safety of these drugs in this new context [47]. To date, this repurposing of established drugs and the development of vaccines has mainly been driven by the common approach of considering SARS-CoV-2 virulence and the host immune reaction as the primary factors in the pathogenesis of COVID-19 [8]. In contrast, according to the triggering role of an ACE2/Ang II imbalance in SARS-CoV-2 pathogenicity [20, 21], we hypothesized that COVID-19 might be attenuated by direct maintenance of innate pulmonary redox, dilation and metabolic functions using a combination of well-tolerated medications via aerosol delivery to human airways. Indeed, we designed and conducted a pilot study of PrEP by using an aerosolized combination of well-tolerated medications (glutathione, inosine and potassium) at low doses in healthy participants at high risk of acquiring SARS-CoV-2 due to their repeated exposure to confirmed COVID-19 cases. Here, we show for the first time the efficacy of pathogenesis-based PrEP for SARS-CoV-2 infection and therefore highlight the following aspects to address COVID-19 prevention and treatment.

In our study, the ACM group experienced no serious adverse events at the chosen doses of 21.3 mg ml/1 glutathione and 8.7 mg ml/1 inosine in hypo-osmolar potassium chloride solution. This is consistent with previous data demonstrating good tolerance of the inhaled components of ACM separately at doses up to an order of magnitude greater in healthy participants or patients [48, 49]. In particular, no toxic or adverse effects were observed for inhaled glutathione at doses of 300 mg or more when administered to participants with pulmonary fibrosis [48]. In addition, when inhaled at concentrations up to 373 μmol (100 mg)/ml, inosine solution had no effect on airway responses in healthy subjects or those with asthma [49]. Therefore, a combination (used simultaneously) of these medications at low doses might be an optimal way to attenuate the main steps of COVID-19 pathogenesis. Importantly, some of the agents can exert a number of physiological effects, increasing the cumulative antiviral efficiency of the combined medication. For instance, in addition to the metabolic activity of inosine, adenosine to inosine modification (A-to-I) by adenosine deaminases acting on RNA is a recently discovered process of posttranscriptional premRNA modification [50]. This process exerts antiviral effects through the incorporation of inosine into double-stranded viral RNA followed by RNase-specific viral degradation [51] as well as through potentiation of immune system sensing [44].

Limitations of our study include the short duration (28 days), the low number of participants (*n* = 99), and the lack of placebo or control medications. Despite planning to recruit healthy volunteers aged 18–80 years, the study included fairly young volunteers. Further research is needed to evaluate pathogenesis-based combination medications for pre- and postexposure prophylaxis of COVID-19 in different populations, including older age groups and individuals with underlying medical conditions, using multicenter randomized placebo-controlled methods.

## Conclusions

The significant reduction in SARS-CoV-2 incidence among HCWs treated with ACM in our study might support the hypothesized importance of the virus-induced ACE2/Ang II regulatory imbalance in the human airways, along with the consequent robust pulmonary hypoxia and peripheral ventilation-perfusion abnormalities induced. Moreover, the results suggest the important prevalence of these processes over the diverse host immune response and viral load, per se, in the pathogenesis of COVID-19. Our approach and findings might be used either to prevent SARS-CoV-2 infection or to support ongoing and new research into more effective treatments for COVID-19.

## Supplementary Information

**Additional file 1.** Protocol of the study MIBVD-19 of 22 April 2020.

## Data Availability

The datasets used and/or analyzed during the current study are available from the corresponding author on reasonable request.

## Abbreviations

COVID-19: Coronavirus disease 2019
SARS-CoV-2: Severe acute respiratory syndrome coronavirus 2
HCWs: Healthcare workers
PrEP: Preexposure prophylaxis
ACE2: Angiotensin-converting enzyme 2
Ang II: Angiotensin II
SMCs: Smooth muscle cells
HPV: Hypoxic pulmonary vasoconstriction
ACM: Aerosolized combination medication
GSH: Glutathione
